# Genetic polymorphisms of *mTOR* and cancer risk: a systematic review and updated meta-analysis

**DOI:** 10.18632/oncotarget.10805

**Published:** 2016-07-24

**Authors:** Jin Zining, Xu Lu, He Caiyun, Yuan Yuan

**Affiliations:** ^1^ Tumor Etiology and Screening Department of Cancer Institute and General Surgery, Key Laboratory of Cancer Etiology and Prevention of Liaoning Provincial Education Department, The First Affiliated Hospital of China Medical University, Shenyang, China; ^2^ Department of Molecular Diagnostics, Sun Yat-Sen University Cancer Center, State Key Laboratory of Oncology in South China, Collaborative Innovation Center for Cancer Medicine, Guangzhou, China

**Keywords:** mTOR, polymorphism, cancer risk, systematic review, meta-analysis

## Abstract

mTOR regulates several cellular processes that are critical for tumorigenesis. However, previous studies on the association of *mTOR* polymorphisms with predisposition to different cancer types are somewhat contradictory. Therefore, we performed a systematic review and updated meta-analysis of the available evidence regarding the relationship between *mTOR* single nucleotide polymorphisms (SNPs) and cancer risk. Up to November 2015, 23 original publications were identified covering 20 *mTOR* SNPs, of which seven SNPs (rs2536, rs2295080, rs1883965, rs1034528, rs17036508, rs3806317 and rs1064261) were included in the final meta-analysis. We estimated the summary odds ratios (ORs) and corresponding 95% confidence intervals (CIs) for *mTOR* polymorphisms and cancer risk, and used the model-free approach to investigate the biological effect of each polymorphism. Our meta-analysis found that rs1883965, rs1034528, and rs17036508 were correlated with increased cancer risk in the complete over-dominant model (rs1883965 GA versus GG/AA: fixed-effects OR=1.15, 95% CI 1.02-1.29; rs1034528 GC versus GG/CC: fixed-effects OR=1.30, 95% CI 1.13-1.48; rs17036508 TC versus CC/TT: fixed-effects OR=1.23, 95% CI 1.06-1.43). Stratifying analyses by cancer type, we found that the rs2295080 G allele was associated with a significantly higher risk of acute leukemia in the recessive model (GG versus GT/TT: fixed-effects OR=2.08, 95% CI 1.34-3.22) and a lower risk of genitourinary cancers in the dominant model (TG/GG versus TT: fixed-effects OR=0.77, 95% CI 0.68-0.86). Interestingly, further expression analysis showed that homozygous variant genotype carriers of rs1883965, rs1034528 and rs17036508 had lower *mTOR* transcript levels, based on HapMap data.

## INTRODUCTION

The mammalian target of rapamycin (mTOR, also known as FRAP1), a key downstream effector of the phosphatidylinositol-3-kinase (PI3K)/AKT signaling pathway, regulates several cellular processes that are critical for oncogenesis, such as cell proliferation, apoptosis, migration, metabolism, and angiogenesis [1-4]. Deregulation of the PI3K pathway is one of the most frequent alterations occurring in human cancer [5]. Unsurprisingly, aberrant expression of *mTOR*, including both over-expression and over-activation, has been observed in lung adenocarcinoma, hepatocellular cancer, large intestine adenocarcinoma, renal cell carcinoma, and extrahepatic cholangiocarcinoma [6-10]. These changes may be caused by genetic alterations [11], and single nucleotide polymorphisms (SNPs) widespread in the human genome [12], have been extensively studied in *mTOR* to identify susceptibility loci for cancer.

Human *mTOR*, located on chromosome 1p36.2, is approximately 156 kb in length and is composed of 59 exons. According to the International HapMap Project Database (http://hapmap.ncbi.nlm.nih.gov/), 181 SNPs in *mTOR* have been reported in different populations, including those from Utah (US residents with ancestry from Europe; CEU), China (Han Chinese in Beijing; CHB), Japan (Japanese in Tokyo; JPT), and Nigeria (Yoruba in Ibadan; YRI). These 181 SNPs are distributed throughout *mTOR*, as well as 5kb upstream and downstream of the gene. Of these, 129 are considered common based on a minor allele frequency ≥ 5%, including seven SNPs in the 5′ upstream region, five SNPs in exonic regions, 112 in intronic regions, and five in the 3′ untranslated region (UTR) and downstream region.

Since Slattery et al. [13] first reported a positive association between *mTOR* rs1057079 and colon cancer risk in 2010, clinical evidence has accumulated regarding the relationship between *mTOR* SNPs and the risk of various cancers, such as gastric cancer [14-18], esophageal carcinoma [19, 20], endometrial cancer [21], renal cell cancer [10, 22], acute leukemia [23, 24], and colorectal cancer [25]. Previously, Shao et al. [26] performed a meta-analysis pooling the data from six case-control studies and indicated an association between rs2295080 in the promoter region of *mTOR* and cancer risk. Since then, eight case-control studies (six original articles and two abstracts) [16-18, 20, 24, 25, 27, 28] have been published that reveal more potentially functional *mTOR* SNPs and challenge conclusions from previous meta-analyses. The evidence is controversial for those *mTOR* SNPs investigated, partially because of insufficient statistical power. Consequently, we performed this updated meta-analysis to reassess the effect of *mTOR* polymorphisms within oncogenesis and to provide a more precise estimation of the associations.

## RESULTS

### Characteristics of eligible studies

**Figure 1:**
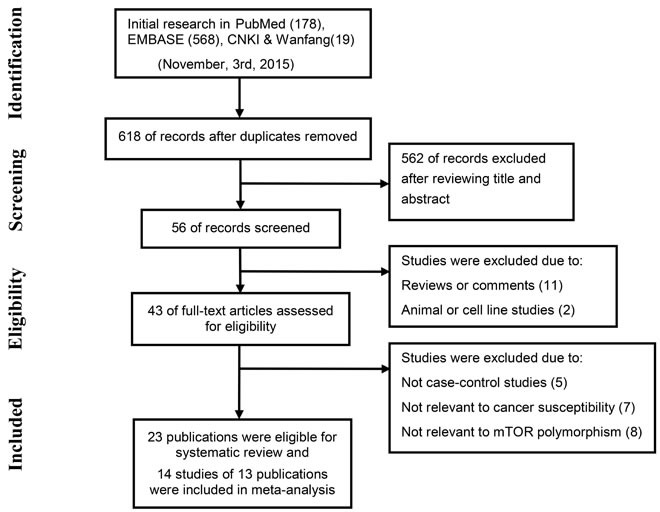
The flow chart shows study selection for this systematic review

The selection process for eligible studies is shown in the flow diagram (Figure 1). A total of 23 case-control studies matched the inclusion criteria [10, 13-25, 27-30, 49, 53, 54, 73, 74], including one that discussed the relationship between *mTOR* polymorphisms and meningioma [28], which is generally considered to be benign. The main characteristics and results of the eligible studies are presented in Supplementary Material. Seven SNPs (rs2536, rs2295080, rs1883965, rs1034528, rs17036508, rs3806317, and rs1064261) included in the final meta-analysis were analyzed in at least two series and were described in 14 studies (one article examined the association in independent populations of childhood acute lymphoblastic leukemia and acute myeloid leukemia, so this was treated as two separate studies). Of the 14 studies, four focused on gastric cancer [14, 15, 17, 18], three on childhood acute leukemia [23, 24], two on prostate cancer [29, 30], two on esophageal carcinoma [19, 20], and one each on hepatocellular carcinoma [31], renal cell cancer [10] and colorectal cancer [25]. All studies were conducted in Asian populations, and genotype distributions among controls were consistent with Hardy-Weinberg equilibrium (HWE). Newcastle-Ottawa Scale (NOS) scores of these studies were higher than 6 (moderate-high quality). Detailed information on the studies included in the meta-analysis is provided in Table 1.

**Table 1:** Main characteristics of studies included in the meta-analysis

First author	Year	Cancer type	Ethnicity	Source of	Sample size	Polymorphism	Quality score	Selection	Comparability	Exposure
			(Country)	control	(case/control)					
Cao, Q. [10]	2012	Renal cell cancer	Asian(China)	HB	710/760	rs2536, rs2295080	8	3	2	3
Chen, J. [29]	2012	Prostate cancer	Asian(China)	HB	666/708	rs2536, rs2295080	7	3	1	3
Huang, L. [23]	2012	ALL	Asian(China)	HB	417/554	rs2536, rs2295080	7	3	2	2
He, J. [14]	2013	Gastric cancer	Asian(China)	PB	1125/1196	rs2536, rs1883965	7	3	1	3
Xu, M. [15]	2013	Gastric cancer	Asian(China)	HB	753/854	rs2295080	6	3	1	2
Mao, L. Q. [49]	2013	Hepatocellular carcinoma	Asian(China)	HB	1048/1052	rs2536, rs1883965	7	3	2	2
Zhu, M. L. [19]	2013	Esophageal carcinoma	Asian(China)	PB	1123/1121	rs2536, rs1883965	8	4	1	3
Li, Q. [30]	2013	Prostate cancer	Asian(China)	PB	1004/1051	rs2536, rs2295080, rs1883965, rs1034528, rs17036508, rs3806317	9	4	2	3
Xu, M. [25]	2015	Colorectal cancer	Asian(China)	HB	737/777	rs2295080	7	3	1	3
Zhu,J.H. [20]	2015	Esophageal carcinoma	Asian(China)	PB	1116/1117	rs2295080, rs1064261	7	4	1	2
Piao, Y. [17]	2015	Gastric cancer	Asian(China)	PB	483/673	rs1064261	6	3	0	3
Wang,M.Y. [18]	2015	Gastric cancer	Asian(China)	HB	1002/1003	rs2295080, rs1034528, rs17036508, rs3806317	6	3	1	2
Zhao, P. [24]	2015	ALL&AML	Asian(China)	HB	180/296	rs2295080	6	2	1	3

### Meta-analysis of *mTOR* rs2536

Seven studies, consisting of 6093 cases and 6442 controls, investigated the association between SNP rs2536 and cancer risk. We carried out a meta-analysis of rs2536 overall and in different cancer types under various genetic models. The seven studies were homogenous for OR1 and OR3, but heterogeneity was significant for OR2 (*I^2^* = 62.4%, p_het_ = 0.014). After excluding Li's study [30], which seemed to be the main source of heterogeneity according to sensitivity analysis, the remaining six studies were homogenous for OR1, OR2, and OR3. rs2536 OR1, OR2, and OR3 were 1.61 (*P* = 0.501), 0.97 (*P* = 0.504), and 1.20 (*P* = 0.422), respectively, suggesting a recessive effect of allele C. Therefore, the TC and TT genotypes were combined and compared with the CC genotype. A non-significant increase in cancer risk for the CC genotype was found (fixed-effect OR = 1.17, 95% CI 0.76-1.80, *P* = 0.485).

Three studies focusing on genitourinary cancers (prostate cancer and renal cell cancer) were homogenous for OR1 and OR3, but heterogeneity was significant for OR2 (*I^2^* = 84.8%, p_het_ = 0.001), which discouraged us from calculating an overall estimate. Digestive system cancers (esophageal carcinoma, gastric cancer and hepatocellular carcinoma) investigated in three studies were homogenous for OR1, OR2, and OR3, but still no significant association was observed. Accordingly, it appeared that rs2536 had no significant effect on susceptibility to cancer (Table 2).

**Table 2:** Non-significant meta-analysis results of the association between mTOR polymorphisms and cancer risk

	No.of studies	OR(95%CI)	*P*	I2(%)	*P*_het_	Model
**rs2536**						
Total	7(6093/6442)					
CC *vs*.TT		1.11(0.75,1.64)	0.613	0	0.921	Fixed-effects model
TC *vs*.TT		1.01(0.86,1.18)	0.902	62.4	0.014	Random-effects model
CC *vs*.TC		1.06(0.71,1.59)	0.764	0	0.667	Fixed-effects model
Excluding Li's study	6(5089/5391)					
CC *vs*.TT		1.16(0.75, 1.79)	0.501	0	0.884	Fixed-effects model
TC *vs*.TT		0.97(0.87,1.07)	0.504	20.4	0.28	Fixed-effects model
CC *vs*.TC		1.20(0.77,1.87)	0.422	0	0.77	Fixed-effects model
CC *vs*.CT/TT		1.17(0.76, 1.80)	0.485	0	0.869	Fixed-effects model
Genitourinary cancers	3(2380/2519)					
CC *vs*.TT		1.01(0.52, 1.98)	0.966	0	0.901	Fixed-effects model
TC *vs*.TT		1.00(0.67, 1.49)	0.991	84.8	0.001	Random-effects model
CC *vs*.TC		0.93(0.47, 1.82)	0.824	0	0.472	Fixed-effects model
Digestive system cancers	3(3296/3369)					
CC *vs*.TT		1.03(0.61, 1.74)	0.927	0	0.905	Fixed-effects model
CT *vs*.TT		1.06(0.93, 1.21)	0.378	0	0.677	Fixed-effects model
CC *vs*.CT		0.98(0.57, 1.68)	0.94	0	0.974	Fixed-effects model
CC *vs*.CT/TT		1.02(0.60, 1.72)	0.947	0	0.918	Fixed-effects model
**rs3806317**						
Total	2(2006/2054)					
GG *vs*.AA		0.79(0.50, 1.26)	0.326	0	0.358	Fixed-effects model
GA *vs*.AA		1.07(0.82, 1.40)	0.61	70.8	0.064	Random-effects model
GG *vs*.GA		0.73(0.45, 1.17)	0.187	0	0.73	Fixed-effects model
**rs1064261**						
Total	2(1599/1790)					
CC *vs*.TT		0.90(0.38, 2.15)	0.82	0	0.556	Fixed-effects model
TC *vs*.TT		1.14(0.95, 1.37)	0.171	8.7	0.295	Fixed-effects model
CC *vs*.TC		0.82(0.34, 1.99)	0.665	0	0.417	Fixed-effects model
TC *vs*.CC/TT		1.14(0.95, 1.37)	0.168	11.9	0.287	Fixed-effects model

### Meta-analysis of *mTOR* rs2295080

Ten studies, consisting of 6585 cases and 7120 controls, investigated the association between SNP rs2295080 and cancer risk. These studies were heterogeneous for OR1 (*I^2^* = 67.7%, p_het_ = 0.001), OR2 (*I^2^* = 65.5%, p_het_ = 0.002) and OR3 (*I^2^* = 49.9%, p_het_ = 0.035). We failed to remove heterogeneity by excluding outliers identified by sensitivity analysis, so studies were grouped by cancer type to explore some possible major sources of heterogeneity (Table 3).

**Table 3:** Significant meta-analysis results of the association between mTOR polymorphisms and cancer risk

	No.of studies	OR(95%CI)	*P*	I2(%)	*P*_het_	Model
**rs2295080**						
Total						
GG *vs*.TT		0.97(0.73, 1.31)	0.86	67.7	0.001	Random-effects model
TG *vs*.TT		0.91(0.80, 1.04)	0.151	65.5	0.002	Random-effects model
GG *vs*.TG		1.04(0.81, 1.32)	0.774	49.9	0.035	Random-effects model
Genitourinary cancers	3(2380/2519)					
GG *vs*.TT		0.76(0.59, 0.99)	**0.045**	0	0.934	Fixed-effects model
TG *vs*.TT		0.77(0.68, 0.87)	**<0.001**	0	0.99	Fixed-effects model
GG *vs*.TG		0.99(0.76, 1.30)	0.955	0	0.92	Fixed-effects model
TG/GG *vs*.TT		0.77(0.68, 0.86)	**<0.001**	0	0.996	Fixed-effects model
Digestive system cancers	4(3608/3751)					
GG *vs*.TT		0.78(0.54, 1.11)	0.169	59.6	0.059	Random-effects model
TG *vs*.TT		0.97(0.80, 1.19)	0.785	76.3	0.005	Random-effects model
GG *vs*.TG		0.81(0.64, 1.02)	0.073	0	0.586	Fixed-effects model
Acute leukemia	3(597/850)					
GG *vs*.TT		2.12(1.36, 3.30)	**0.001**	25.1	0.263	Fixed-effects model
TG *vs*.TT		1.06(0.86, 1.33)	0.578	0	0.691	Fixed-effects model
GG *vs*.TG		2.00(1.26, 3.17)	**0.003**	37.5	0.202	Fixed-effects model
GG *vs*.GT/TT		2.08(1.34, 3.22)	0.001	33	0.225	Fixed-effects model
**rs1883965**						
Total	4(4300/4420)					
AA *vs*.GG		0.91(0.54, 1.54)	0.733	49.6	0.114	Fixed-effects model
GA *vs*.GG		1.15(1.02, 1.29)	**0.019**	0	0.484	Fixed-effects model
AA *vs*.GA		0.79(0.46, 1.36)	0.399	41.3	0.164	Fixed-effects model
GA *vs*.GG/AA		1.15(1.02, 1.29)	**0.018**	0	0.514	Fixed-effects model
Digestive system cancers	3(3296/3369)					
AA *vs*.GG		0.77(0.24, 2.46)	0.059	65.2	0.056	Random-effects model
GA *vs*.GG		1.18(1.03, 1.35)	0.014	0	0.415	Fixed-effects model
AA *vs*.GA		0.66(1.23, 1.91)	0.447	56.5	0.1	Random-effects model
**rs1034528**						
Total	2(2006/2054)					
CC *vs*.GG		0.95(0.66,1.38)	0.791	0	0.484	Fixed-effects model
GC *vs*.GG		1.30(1.13, 1.48)	**<0.001**	0	0.892	Fixed-effects model
CC *vs*.GC		0.73(0.50, 1.07)	0.109	0	0.526	Fixed-effects model
GC *vs*.GG/CC		1.30(1.13, 1.48)	**<0.001**	0	0.951	Fixed-effects model
**rs17036508**						
Total	2(2006/2054)					
CC *vs*.TT		0.99(0.64, 1.55)	0.975	0	0.808	Fixed-effects model
TC *vs*.TT		1.23(1.06, 1.43)	**0.006**	0	0.959	Fixed-effects model
CC *vs*.TC		0.81(0.51, 1.28)	0.36	0	0.8	Fixed-effects model
TC *vs*.CC/TT		1.23(1.06, 1.43)	**0.006**	0	0.945	Fixed-effects model

The results are in bold if *P* < 0.05.

**Figure 2:**
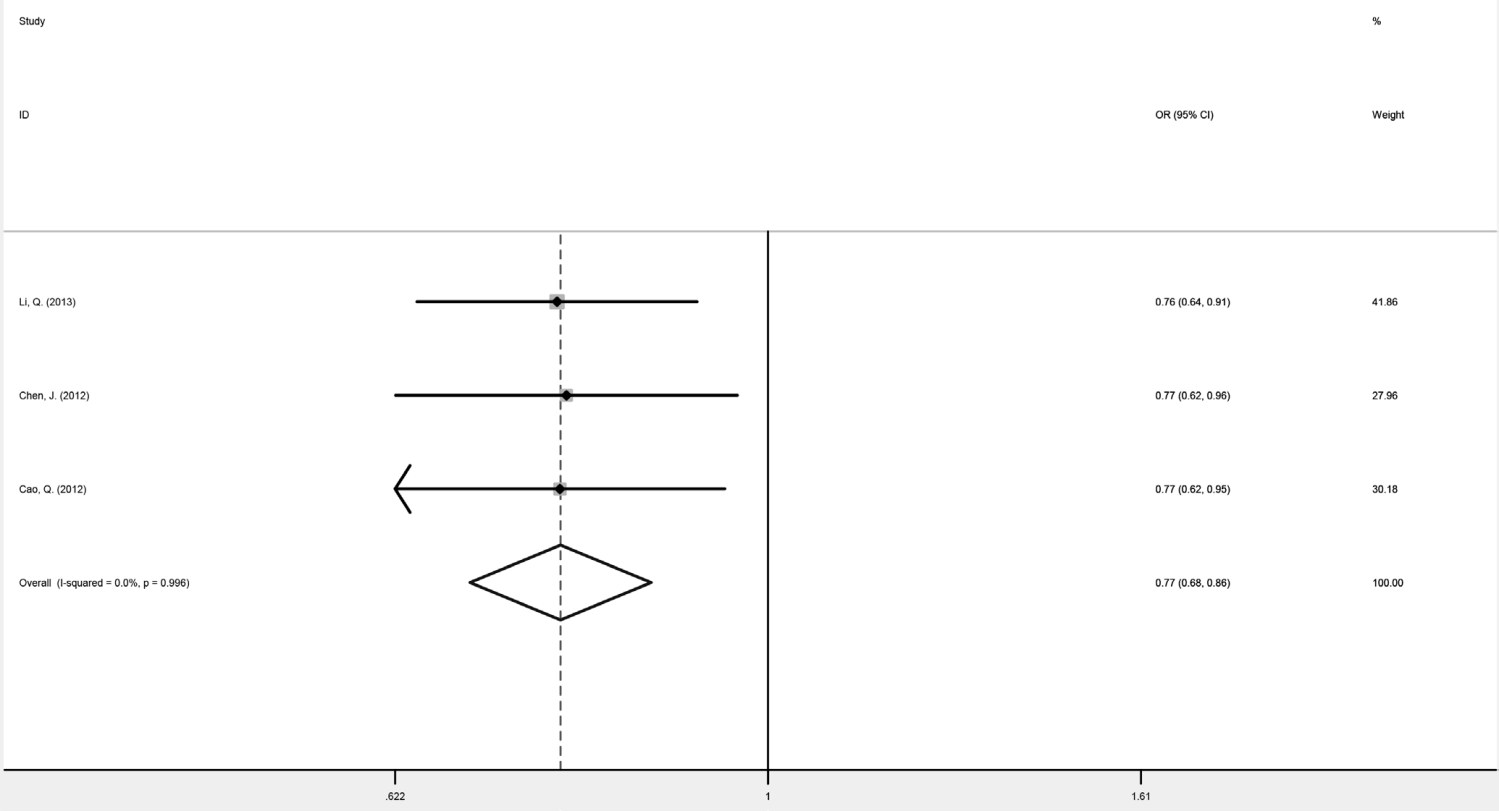
Forest plots of fixed-effects ORs for *mTOR* rs2295080 and risk of genitourinary cancers based on a dominant model (TG/GG *versus* TT)

**Figure 3:**
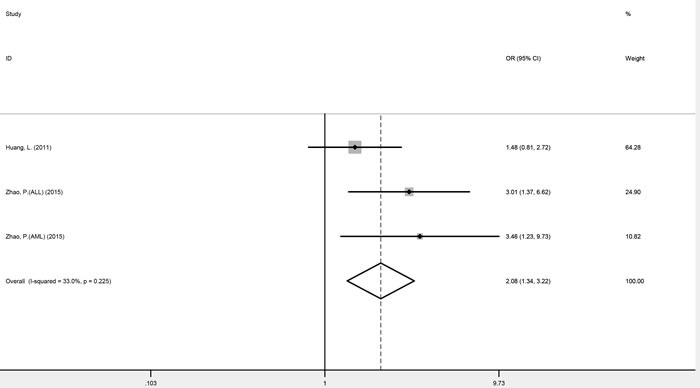
Forest plots of fixed-effects ORs for *mTOR* rs2295080 and risk of acute leukemia based on a recessive model (GG *versus* GT/TT)

Three studies focusing on genitourinary cancers (prostate cancer and renal cell cancer) were homogenous for OR1, OR2, and OR3, which were 0.76 (*P* = 0.045), 0.77 (*P* < 0.001), and 0.99 (*P* = 0.955), respectively, suggesting a dominant effect of the G allele. Therefore compared with the TT genotype, carriers of the G allele (GG and TG genotypes) were shown to have a significantly reduced cancer risk (fixed-effect OR = 0.77, 95% CI 0.68-0.86, *P* < 0.001) (Figure 2). Digestive system cancers (esophageal carcinoma, gastric cancer and colorectal cancer) were investigated in four studies. While these were homogenous for OR3, heterogeneity was significant for OR1 (*I^2^* = 59.6%, p_het_ = 0.059) and OR2 (*I^2^* = 76.3%, p_het_ = 0.005). Consequently, there was no indication to pool the estimates. Childhood acute leukemia (acute lymphoblastic leukemia and acute myeloid leukemia), a non-solid cancer, was investigated in three studies, which appeared homogeneous and suggested a recessive model (GG *versus* GT/TT: fixed-effect OR = 2.08, 95% CI 1.34-3.22, *P* = 0.001) (Figure 3) (Table 3).

### Meta-analysis of *mTOR* rs1883965

**Figure 4:**
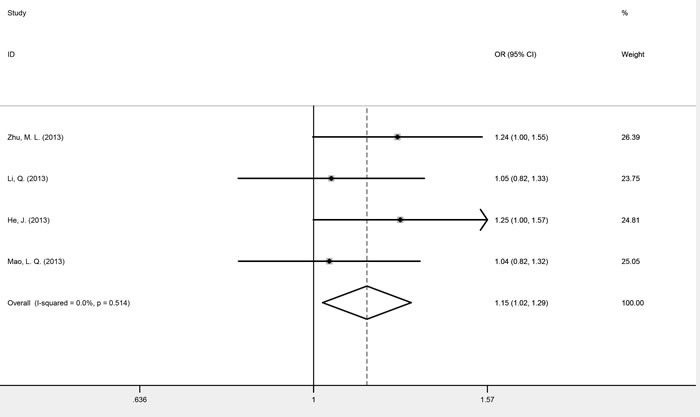
Forest plots of fixed-effects ORs for *mTOR* rs1883965 and cancer risk based on a complete over-dominant model (GA *versus* GG/AA)

Four studies, consisting of 4300 cases and 4420 controls, investigated the association between SNP rs1883965 and cancer risk. These studies were homogenous for OR1, OR2, and OR3, which were 0.91 (*P* = 0.733), 1.15 (*P* = 0.019), and 0.79 (*P* = 0.399), respectively. The summary estimate under the heterozygous model (GA *versus* GG) was statistically significant, implying that carriers of the rs1883965 GA heterozygote were more susceptible to cancer development. A comparison of GA *versus* GG/AA genotypes confirmed a complete over-dominant model (fixed-effect OR = 1.15, 95% CI 1.02-1.29, *P* = 0.018), with the GA heterozygote having a higher cancer risk than either GG or AA homozygotes (Figure 4 and Table 3).

Three studies focused on digestive tract cancers (esophageal carcinoma, gastric cancer, and hepatocellular carcinoma). Heterogeneity was noted for OR1 (*I^2^* = 65.2%, p_het_ = 0.056), so no further analysis was conducted.

### Meta-analysis of *mTOR* rs1034528, rs1064261, rs17036508, and rs3806317

Two studies determined the association between SNPs rs1034528, rs17036508, and rs3806317 and cancer risk, with 2,006 cancer patients and 2,054 controls enrolled. For rs1034528, studies were homogenous for OR1, OR2, and OR3, with values of 0.95 (*P* = 0.791), 1.30 (*P* < 0.001), and 0.73 (*P* = 0.109), respectively, suggesting a complete over-dominant model (GC *versus* GG/CC: fixed-effect OR = 1.30, 95% CI 1.13-1.48, *P* < 0.001). For rs17036508, heterogeneity tests were also negative for OR1, OR2, and OR3, at 0.99 (*P* = 0.975), 1.23 (*P* = 0.006), and 0.81 (*P* = 0.36), respectively, again suggesting a complete over-dominant model (GC *versus* GG/CC: fixed-effect OR = 1.23, 95% CI 1.06-1.43, *P* = 0.006) (Table 3). For rs3806317, heterogeneity tests were negative for OR1 and OR3 but significant for OR2 (*I^2^* = 70.8%, p_het_ = 0.064), so it was not appropriate to meta-analyze the data. Two studies reporting rs1064261 genotype data from 1599 cancer patients and 1790 controls were homogenous for OR1, OR2, and OR3, with values of 0.90 (*P* = 0.82), 1.14 (*P* = 0.171), and 0.82 (*P* = 0.665), respectively. These estimates indicated a complete over-dominant model, whereas the overall gene effect was not significant (TC *versus* CC/TT: OR = 1.14, 95% CI 0.95-1.37, *P* = 0.287) (Table 2).

### Publication bias

No obvious publication bias was detected, with the exception of rs1883965, which exhibited slight publication bias (Egger's test: *P* = 0.084; Table 4).

**Table 4:** Publication bias

	Begg's test			Egger's test	
	***z* value**	***p* value**		***t* value**	***p* value**
**rs2295080**					
Genitourinary cancers					
TG/GG *vs*.TT	1.57	0.117		1.72	0.335
Acute leukemia					
GG *vs*.GT/TT	0.52	0.602		2.26	0.265
**rs1883965**					
GA *vs*.GG/AA	-0.68	0.497		-3.24	0.084
**rs17036508**					
TC *vs*.CC/TT	-1	0.317		/	/
**rs1034528**					
GC *vs*.GG/CC	-1	0.317		/	/

### Bioinformatics and expression analysis

Among the *mTOR* SNPs analyzed, data from the SNPinfo database suggested that six (rs3806317, rs1034528, rs12125777, rs1883965, rs2295080, and rs1074078) are located in transcription factor binding sites (TFBS), four (rs11121691, rs1057079, rs17036508, and rs1064261) may affect exonic splicing enhancer or exonic splicing silencer binding site activity or even abolish a protein domain, and two (rs2536 and rs17036508) are within microRNA (miRNA) binding sites.

As for the *mTOR* SNPs included in this meta-analysis, the F-SNP (FS) database found no functional information for the 3′-UTR SNP rs2536. Intronic polymorphisms rs3806317, rs17036508, and rs1883965 had an FS score of 0.101, while that of rs1034528 was 0.398, probably reflecting the frameshift coding changes it may cause. As a synonymous coding polymorphism, rs1064261 was shown to be conserved across multiple species with an FS score of 0.33. The *mTOR* promoter SNP rs2295080 was found to have an FS score of 0.101. Detailed information on the SNP functional bioinformatics analysis is shown in Table 5.

**Table 5:** Bioinformatics analysis of investigated *mTOR* SNPs by using SNPinfo database and F-SNP database

rs	Position	Region	Allele	TFBS	Splicing	Splicing	miRNA	miRNA	RegPotential	Conservation	F-SNP	F-SNP	Nearby Gene	Distance (bp)
					(ESE or ESS)	(abolish domain)	(miRanda)	(Sanger)			Functional Category	FS score		
rs2536	11089300	3'-UTR	C/T	--	--	--	Y	Y	0.026074	0.481	not known	/	FRAP1	121||155853
rs11121691	11103914	exon	C/T	--	Y	--	--	--	0.46852	1	synonymous coding	0.195	FRAP1	14735||141239
											splicing_regulation			
											transcriptional_regulation			
rs12732063	11113819	intron	A/G	--	--	--	--	--	0	0	not known	/	FRAP1	24640||131334
rs1057079	11127645	exon	T/C	--	Y	Y	--	--	0.34945	0.998	synonymous coding	0.33	FRAP1	38466||117508
											splicing_regulation			
											transcriptional_regulation			
rs1770345	11137167	intron	A/C	--	--	--	--	--	0	0.003	transcriptional_regulation	0.101	FRAP1	47988||107986
rs11585553	11156401	intron	A/G	--	--	--	--	--	0	0	not known	/	FRAP1	67222||88752
rs11121696	11160650	intron	C/T	--	--	--	--	--	0.151757	0.001	not known	/	FRAP1	71471||84503
rs3806317	11170803	intron	G/A	Y	--	--	--	--	0	0	transcriptional_regulation	0.101	FRAP1	81624||74350
rs1034528	11171719	intron	C/G	Y	--	--	--	--	0.105946	0.28	transcriptional_regulation	0.398	FRAP1	82540||73434
rs17036508	11178621	intron	C/T	--	Y	--	Y	--	0.117154	0	transcriptional_regulation	0.101	FRAP1/ANGPTL7	89442/6636||66532/4
rs1010447	11192383	intron	C/T	--	--	--	--	--	NA	0	transcriptional_regulation	0.101	FRAP1	103204||52770
rs12116957	11196005	intron	G/T	--	--	--	--	--	0.093901	0	transcriptional_regulation	0.101	FRAP1	106826||49148
rs12124983	11208765	intron	C/T	--	--	--	--	--	0	0.566	transcriptional_regulation	0.101	FRAP1	119586||36388
rs1064261	11211345	exon	G/A	--	Y	Y	--	--	0.429961	1	synonymous coding	0.33	FRAP1	122166||33808
											splicing_regulation			
											transcriptional_regulation			
											conserved			
rs718206	11217061	intron	A/T	--	--	--	--	--	0	0	not known	/	FRAP1	127882||28092
rs2024627	11221377	intron	C/T	--	--	--	--	--	NA	0.001	not known	/	FRAP1	132198||23776
rs12125777	11244252	intron	C/T	Y	--	--	--	--	0	0.005	not known	/	FRAP1	155073||901
rs1883965	11244743	intron	A/G	Y	--	--	--	--	0	0	transcriptional_regulation	0.101	FRAP1	155564||410
rs2295080	11245215	promoter	G/T	Y	--	--	--	--	0.284745	0	transcriptional_regulation	0.101	ANGPTL7||UBIAD1	-66590||-10651
rs1074078	11249375	5' near gene	C/T	Y	--	--	--	--	NA	0	not known	/	ANGPTL7||UBIAD1	-70750||-6491

No content is listed under non-synonymous polymorphisms (nsSNPs), Stop Codon, and Polyphen

Given that rs2295080, rs1883965, rs1034528 and rs17036508 showed a potential association with cancer susceptibility, we further explored their relationship with *mTOR* transcript expression levels using the SNPexp web tool (Table 6-7). No significant alteration in transcript expression was observed for rs2295080. In the YRI population, the expression level of the rs1883965 heterozygote was lower than that of homozygotes (complete over-dominant: *P* = 0.043) (Table 6). For rs1034528, the C allele was correlated with significantly decreased levels of *mTOR* transcript expression compared with the G allele in both the European population (heterozygous: *P* = 0.023; dominant: *P* = 0.035; complete over-dominant: *P* = 0.019) and all populations (heterozygous: *P* = 0.002; dominant: *P* = 0.001; complete over-dominant: *P* = 0.003). A similar trend was observed for rs17036508 for all populations (heterozygous: *P* = 0.001; dominant: *P* = 0.001; complete over-dominant: *P* = 0.001), although no significant linkage disequilibrium was found between these two polymorphisms among the four different ethnicities (Table 7).

**Table 6:** *MTOR* expression analysis by the genotypes of rs2295080 and rs1883965, using data from the HapMap

	rs2295080					rs1883965				
Ethnicities	Genotypes	No.	Mean±SD	P^b^	P^c^	Genotypes	No.	Mean±SD	P^b^	P^c^
CEU	TT	41	8.55±0.18		0.122	GG	35	8.59±0.18		0.105
	TG	42	8.45±0.29	0.068		GA	40	8.51±0.28	0.158	
	GG	7	8.48±0.25	0.367		AA	6	8.47±0.21	0.13	
	Dominant	49	8.45±0.28	0.068		Dominant	46	8.50±0.27	0.114	
	Complete	48	8.54±0.19	0.09		Complete	41	8.57±0.19	0.255	
	over-dominant					over-dominant				
Asian	TT	NA	/	/	/	GG	66	8.22±0.24		/
	TG	NA	/	/		GA	19	8.18±0.26	0.494	
	GG	NA	/	/		AA	0	/	/	
	Dominant	NA	/	/		Dominant	/	/	/	
	Complete	NA	/	/		Complete	/	/	/	
	over-dominant					over-dominant				
YRI	TT	NA	/	/	/	GG	4	8.33±0.31		0.304
	TG	NA	/	/		GA	36	8.15±0.26	0.203	
	GG	NA	/	/		AA	43	8.26±0.24	0.586	
	Dominant	NA	/	/		Dominant	40	8.17±0.26	0.108	
	Complete	NA	/	/		Complete	47	8.26±0.24	**0.043**	
	over-dominant					over-dominant				
All	TT	41	8.55±0.18		0.122	GG	105	8.36±0.26		0.237
	TG	42	8.45±0.29	0.068		GA	95	8.31±0.29	0.185	
	GG	7	8.48±0.25	0.367		AA	49	8.32±0.21	0.304	
	Dominant	49	8.45±0.28	0.068		Dominant	144	8.31±0.27	0.149	
	Complete	48	8.54±0.19	0.09		Complete	154	8.34±0.24	0.272	
	over-dominant					over-dominant				

The results are in bold if *P* < 0.05.

* Genotyping data and transcript expression levels for mTOR by genotypes were from the HapMap phase II release 23 data (rs2295080, rs1883965)

# Two-tailed Student's *t* test

^ *P* values for the trend test of *mTOR* transcript expression among 3 genotypes for each SNP from a general linear model

**Table 7:** *MTOR* expression analysis by the genotypes of rs1034528 and rs17036508, using data from the HapMap

	rs1034528					rs17036508				
Ethnicities	Genotypes	No.	Mean±SD	P^b^	P^c^_trend_	Genotypes	No.	Mean±SD	P^b^	P^c^_trend_
CEU	GG	48	8.59±0.19		0.107	TT	81	8.54±0.24	/	/
	GC	35	8.47±0.29	**0.023**		TC	0	/	/	
	CC	4	8.60±0.19	0.937		CC	0	/	/	
	Dominant	39	8.48±0.28	**0.035**		Dominant	/	/	/	
	Complete	52	8.59±0.18	**0.019**		Complete	/	/	/	
	over-dominant					over-dominant				
Asian	GG	59	8.24±0.23		0.221	TT	71	8.21±0.24		0.682
	GC	29	8.18±0.25	0.339		TC	13	8.23±0.23	0.805	
	CC	1	8	0.31		CC	1	7.9	0.205	
	Dominant	30	8.18±0.25	0.278		Dominant	14	8.21±0.24	0.937	
	Complete	60	8.23±0.23	0.367		Complete	72	8.21±0.24	0.76	
	over-dominant					over-dominant				
YRI	GG	21	8.28±0.22		0.646	TT	47	8.24±0.24		0.372
	GC	44	8.16±0.26	0.075		TC	35	8.15±0.26	0.09	
	CC	23	8.24±0.23	0.575		CC	2	8.46±0.28	0.227	
	Dominant	65	8.20±0.25	0.488		Dominant	37	8.17±0.27	0.159	
	Complete	44	8.26±0.23	0.059		Complete	49	8.25±0.24	0.064	
	over-dominant					over-dominant				
All	GG	128	8.39±0.25		**0.01**	TT	199	8.36±0.26		**0.002**
	GC	108	8.28±0.27	**0.002**		TC	48	8.22±0.24	**0.001**	
	CC	28	8.32±0.23	0.162		CC	3	8.26±0.36	0.535	
	Dominant	136	8.29±0.26	**0.001**		Dominant	51	8.22±0.24	**0.001**	
	Complete	156	8.37±0.24	**0.003**		Complete	202	8.36±0.26	**0.001**	
	over-dominant					over-dominant				

The results are in bold if *P* < 0.05.

* Genotyping data and transcript expression levels for mTOR by genotypes were from the HapMap phase II release 23 data (rs1034528) and HapMap phase III release 3 data (rs17036508)

# Two-tailed Student's *t* test

^ *P* values for the trend test of *mTOR* transcript expression among 3 genotypes for each SNP from a general linear model

## DISCUSSION

As the central controller of cellular growth and proliferation, mTOR induces several anabolic processes such as protein synthesis [32], lipogenesis [33, 34] and nucleotide biosynthesis [35, 36], suppresses catabolic processes such as autophagy [37] and lysosome biogenesis [38], and regulates whole body energy metabolism [39] by forming two distinct multiprotein complexes, mTORC1 and mTORC2. Blocking mTOR activation by an mTOR inhibitor, such as everolimus or temsirolimus, exhibits an anti-neoplastic effect and is approved by the Food and Drug Administration and the European Medicines Agency for the treatment of limited types of cancers [40]. As well as their effect on cancer which had developed already, mTOR inhibitors play even more significant roles in cancer prevention [41]. Using sirolimus after renal transplantation could reduce the risk of malignancies for transplant recipients, who are at higher risk of cancer because of immunosuppression [42, 43]. Additionally, rapamycin has been shown to delay carcinogenesis and prolong lifespan in p53-deficient mice [44, 45]. This indicates that mTOR has a critical role to play in oncogenesis. As the mTOR pathway is a central controller of cellular growth, excessive activation caused by mutations or other changes in upstream pathways confers a growth advantage to cancer cells. But beyond that, Overstimulation of the mTOR pathway also accelerates organismal aging and then contributes to oncogenesis indirectly [46].

Genetic alterations are widespread throughout *mTOR* and influence protein function by changing gene expression. Mutant mTOR proteins caused by point mutations around the kinase domain of mTOR demonstrate constitutive activation [11, 47, 48], and have been shown to affect cell cycle progression and cell size in human cancers [11]. Polymorphisms occur more frequently than mutations and are stably inherited within a population. Therefore, determining their impact on the mTOR protein and oncogenesis is of great importance.

The present systematic review evaluated the overall effect of *mTOR* polymorphisms on cancer risk, and the updated meta-analysis evaluated the most commonly investigated *mTOR* polymorphisms: rs2536, rs2295080, rs1883965, rs1034528, rs17036508, rs3806317 and rs1064261. We identified a significant correlation between heterozygotes of SNPs rs1883965, rs1034528 and rs17036508 and increased cancer risk compared with homozygotes. Significant results were also identified for SNP rs2295080 in the subgroup of genitourinary cancers and acute leukemia. No clear associations between the other meta-analyzed polymorphisms and cancer risk were observed.

### SNP rs2536: a controversial association with cancer risk

The rs2536 (T > C) polymorphism in the *mTOR* 3′-UTR was predicted to affect miRNA-binding site activity based on the SNPinfo database. Li et al. [31] previously reported that co-transfection of the rs2536 G allele and A allele with miR-767-3p exhibited different promoter activities. However, previous studies regarding the relationship between rs2536 and cancer risk are inconsistent. In a study by Li et al. [30], rs2536 was correlated with an increased risk of prostate cancer, while an earlier Chinese case-control study [23] focusing on the risk of childhood acute lymphoblastic leukemia reported the opposite effect. Other case-control studies concentrating on esophageal carcinoma [19], gastric cancer [14], prostate cancer [29], renal cell cancer [10], hepatocellular carcinoma [49], and meningioma [28] found no significant association, nor did a previous meta-analysis [26].

The present pooled analysis also found no significant association between rs2536 and cancer risk, after removing the main source of heterogeneity and performing stratified analyses by cancer type. However, several studies indicated that although the main effect was not obvious, rs2536 was included in the best model to predict the risk of esophageal carcinoma [19] and prostate cancer [30], together with polymorphisms known to be associated with cancer susceptibility and environmental factors such as body mass index (BMI). This means that rs2536 should not be simply categorized as “not important”, because it may interact with environmental factors or other genetic variations and is linked to cancer development through joint effects.

### SNP rs2295080: inconsistent roles in genitourinary cancers and acute leukemia

The rs2295080 (T > G) polymorphism located in the *mTOR* upstream region was predicted to be within a TFBS by the SNPinfo database, which has been further confirmed by the lower nuclear protein binding activity of the G allele in human gastric cancer cell line SGC-7901 [15]. Moreover, patients with gastric cancer [15] and renal cell cancer [10] carrying the rs2295080 G allele showed decreased *mTOR* mRNA levels compared with those with the wild-type T allele. Additionally, rs2295080 has been linked to decreased *mTOR* promoter activity in several cell lines [10, 15, 25]. Many previous studies have shown that the rs2295080 G allele is associated with a decreased risk of gastric cancer [15], colon cancer [25], prostate cancer [29, 30], and renal cell cancer [10], and this was supported by a previously published meta-analysis [26]. However, recently, some opposite findings were reported in gastric cancer [18], esophageal carcinoma [20], and childhood acute leukemia [24].

We found that the rs2295080 G allele was associated with a significantly lower risk of genitourinary cancers in the dominant model, and a higher risk of acute leukemia in the recessive model. Therefore, the biological effect of rs2295080 might be cancer-specific. Notably, an obvious divergence of rs2295080 effects was observed in cancers of the digestive system, which might be partially explained by a high degree of heterogeneity, especially for gastric cancer [50]. A more detailed classification based on clinical, histologic, and molecular features will help to elucidate the relationship between rs2295080 and gastrointestinal cancers.

### SNP rs1883965: increased cancer risk under the complete over-dominant model

The SNP rs1883965 (G > A) is located within the first intron of *mTOR*, so is more likely to be involved in the regulation of transcription and be associated with disease compared with SNPs in other introns [51]. The SNPinfo database indicated that rs1883965 is located in a TFBS, which may affect the level or timing of gene expression. Two previous studies indicated increased associations between the rs1883965 A allele and the risks of esophageal carcinoma [19] and gastric cancer [14], and our present meta-analysis found the heterozygote GA to be significantly associated with increased cancer risk compared with homozygotes GG and AA. Interestingly, we observed slightly decreased *mTOR* mRNA expression in YRI individuals carrying the rs1883965 heterozygote GA (*P* = 0.043). Such discrepant results may not be entirely attributed to racial differences, but could reflect the small sample size of gene expression data, which increases the probability of false-positive findings. Therefore, more studies are essential to obtain a more reliable conclusion regarding the association between rs1883965 and *mTOR* transcription.

### SNPs rs1034528 and rs17036508: associated with increased cancer risk under the complete over-dominant model

SNPs rs1034528 (G > C) and rs17036508 (T > C) are both located within intronic regions of *mTOR*. Bioinformatics analysis revealed that rs1034528 is located within a TFBS and causes a frameshift coding change. Its FS score of 0.398 is the highest among those *mTOR* SNPs investigated. SNP rs17036508 was predicted to be located within a miRNA binding site and an exonic splicing enhancer or silencer motif, affecting the splicing of pre-RNA. Previously, the associations between rs1034528 and rs17036508 and gastric and prostate cancer were investigated and the rs1034528 C allele was shown to be a risk factor in two independent studies [18, 30]. In the present study, we found that heterozygote carriers of rs1034528 and rs17036508 were more likely to develop cancer compared with homozygotes. However, expression analysis using HapMap data indicated that homozygotes had higher *mTOR* transcript expression levels. Because the mTOR signaling pathway usually promotes oncogenesis, this finding is unexpected but could be explained by the fact that mTOR mutants caused by different amino acid substitutions have different abilities to phosphorylate substrates S6K1 and 4E-BP1, even though they are expressed at similar levels after nutrient starvation [11]. Thus, it is conceivable that a frameshift within the coding region (rs1034528) or splicing variants (rs17036508) confers hyperactivation to the mTOR protein, which promotes the development of cancer regardless of the expression level. Alternatively, rs1034528 and rs17036508 are respectively located in 5′ upstream region and 3′-UTR of the angiopoietin-like 7 gene (*ANGPTL7*), itself within intron 28 of *mTOR*. *ANGPTL7* expression was reported to be up-regulated by hypoxia in cancer cells and to exert a pro-angiogenetic effect, which is essential in the early stages of tumor development [52]. Therefore, these two polymorphisms might mediate tumor formation by regulating the expression of *mTOR* and *ANGPTL7* simultaneously.

### Other *mTOR* polymorphisms: further investigation required

rs1064261 (T > C) and rs1057079 (A > G) are synonymous SNPs within exonic regions. Although not altering amino acid sequences, they were predicted to interrupt the exonic splicing enhancer or silencer motif, or even abolish a protein domain. Positive correlations of the rs1064261 C allele with an increased risk of neuroendocrine tumors [53] and gastric cancer in men [17] have been reported, while its interaction with rs2295080 was also identified in esophageal squamous cell carcinoma [20]. However, our previous work found no association between rs1064261 and total or phosphorylated mTOR protein in gastric cancer mucosa [17]. The present meta-analysis also revealed no association between rs1064261 and overall cancer risk. As for SNP rs1057079, carriers of the G allele were at higher risk of developing colon cancer and breast cancer [13, 54] and ethnic differences might exist for the effect of rs1057079 on cancer risk [54].

Although several intronic SNPs were excluded from the present meta-analysis and the integrated results of their relationships with cancer risk were not discussed because of the limited number of studies, some of them exhibited potential biological activity. SNPs rs12125777 (C > T) and rs12124983 (C > T) were identified to interfere with transcriptional regulation by bioinformatic analysis; these predictions were confirmed by clinical evidence [27, 53] and so warrant further research. While no functional information currently exists for SNPs rs2024627 (C > T) and rs718206 (A > T), they were shown to be significantly associated with increased colon cancer risk by a case-control study [13]. These SNPs might not be causative of disease but could exist in high linkage disequilibrium with other functional SNPs.

### Gene-gene and gene-environment interactions

Integrated risk estimates of clinical evidence reveal that the *mTOR* SNPs studied to date only have a mild effect on cancer development. The FS integrative scoring system also defined these polymorphisms as having relatively moderate deleterious effects, because no *mTOR* SNPs have yet been assigned an FS score as high as 0.5, which is the median score of disease-related SNPs [55].

Although the magnitude of the effect of an individual *mTOR* SNP on cancer susceptibility appears to be weak, its interaction with functionally relevant variants and environmental factors might have a greater effect on oncogenesis. Carriers of combined risk alleles have been previously shown to have a significantly increased risk of developing various types of cancer, mostly in a dose-dependent manner. For example, Li et al. [30] found that individuals carrying four adverse genotypes from six *mTOR* polymorphisms (rs2536, rs1883965, rs1034528, rs17036508, rs3806317, and rs2295080) exhibited a higher susceptibility of developing prostate cancer (adjusted OR = 1.74, 95% CI 1.20-2.51) compared with those with one or zero adverse genotypes. Such cumulative effects were also observed across SNPs in *mTOR* and mTOR pathway-related genes (*PIK3R1*, *AKT2*, and *PTEN*) [10, 20, 29], or in genes encoding components of mTOR complex 1 (*mLST8* and *RPTOR*) [19]. Several studies assessed *mTOR* haplotype effects on cancer risk [13, 14, 20, 30, 49]. Zhu et al. [20] explored the relationships between haplotypes of rs2295080, rs1064261 and rs1057079 and the risk of esophageal squamous cell carcinoma. Although no significant association was observed when SNPs were analyzed individually, there were clear associations between three of the seven identified haplotypes and increased cancer risk compared with the most frequent haplotype. Interactions of rs2295080 with either rs1057079 or rs1064261 were also found.

Possible interactions between *mTOR* polymorphisms and environmental factors such as smoking status, drinking status, age, sex and BMI have also been reported. Both the effects of individual SNPs and combined risk genotypes were greater in some high-risk subgroups for many cancers such as older adults and smokers [14-16, 18-20, 25, 29, 30]. Furthermore, statistical gene-environment interactions between *mTOR* SNPs and BMI were verified in esophageal squamous cell carcinoma [20] and breast cancer [27]. It has been reported in mouse heart tissue that phosphorylated S6, which reflects the activity of mTOR, is positively related to body weight [56]. Because the mTOR pathway regulates energy metabolism and as cancer can be regarded as a metabolic disorder, it is not surprising that BMI might be a mediator between *mTOR* variants and cancer susceptibility. However, the underlying mechanisms may vary depending on cancer type and remain to be investigated.

### Limitations

A number of limitations of this systematic review and meta-analysis should be noted. First, although we collected all published clinical evidence investigating *mTOR* SNPs and cancer risk, the pooled sample size of this meta-analysis was still relatively small, especially for rs1034528, rs17036508, rs1064261, and rs3806317. This weakened the statistical power and limited our ability to perform more accurate subgroup analyses for specific cancer types. Second, the ethnicity of all available studies for meta-analysis was Han Chinese, so our findings may not be applicable to other populations. However, because Han Chinese is the largest ethnic group in the world and as the gene pool reflects a long history of immigrations and intermarriages with other ethnic groups [57], these data represent the complexity of the impact of *mTOR* polymorphisms on cancer development. Third, all studies included in the systematic review were published in English or Chinese, yet publications in other languages may contain different relevant studies. This may be the main source of publication bias in our meta-analysis. Finally, although the SNPinfo database and F-SNP aim to reduce the number of false-positive results, computational predictions of certain SNPs are only estimates and should be confirmed by functional studies.

### Future directions

The study of *mTOR* polymorphisms has mainly focused on cancer susceptibility in recent years. To date, only 20 of 129 common SNPs within *mTOR* have been investigated in relation to cancer risk. Future studies may benefit from genotyping additional polymorphisms to identify more functionally significant variants. Additionally, although the risk effects of the *mTOR* SNPs so far examined are too small to be regarded as clinically useful, their interactions with other genetic variants or environmental factors have been shown to contribute to further increases in cancer risk either additively or synergistically. The mechanisms of these joint effects deserve further research. *mTOR* polymorphisms may also be associated with clinical outcomes and response to chemoradiotherapy. Several polymorphisms, such as rs2295080, rs11121704, and rs12139042, have been shown to be significantly associated with lung and esophageal cancer [58-60], but more attention should be given to the association of *mTOR* polymorphisms with treatment response to inhibitors of the mTOR pathway. *In vitro* functional studies should also be conducted to confirm these functional predictions and reveal the underlying molecular mechanisms behind the observed associations. Finally, some investigations have found that mTOR inhibitors are able to prolong lifespan not just by inhibiting the growth of tumors, but by postponing the aging process [41, 61]. This means that *mTOR* polymorphisms may also play roles in other age-related diseases, such as cardiovascular and neurodegenerative disorders, and further investigation is required.

## MATERIALS AND METHODS

This systematic review and meta-analysis was conducted following the PRISMA (Preferred Reporting Items for Systematic Reviews and Meta-Analyses) recommendations [62].

### Search strategy

We searched the PubMed, Embase, Chinese National Knowledge Infrastructure and Wanfang Data databases to identify potentially relevant studies published before November 3, 2015, without language restrictions. The following keywords were used jointly as search terms: “mTOR” or “FRAP” or “RAFT1” or “RAPT1”, “polymorphism” or “variant” or “mutation” and “cancer” or “tumor” or “carcinoma” or “carcinoma” or “malignancy”. The full electronic search strategy for PubMed is shown in Appendix 2. We also manually searched the reference lists to identify other potential articles. If overlapping data by the same first author were found, the article with the largest number of subjects was included.

### Inclusion and exclusion criteria

Studies were eligible if they met the following criteria: (1) evaluated the association between *mTOR* polymorphisms and cancer risk; (2) written in English or Chinese; and (3) case-control studies. Studies were excluded if they were: (1) reviews or comments; or (2) animal or cell line studies. Eligible studies were determined by two researchers (ZNJ and LX) independently. Disagreement was resolved by discussion or consulting another researcher (CYH).

### Data extraction and quality assessment

Relevant information, including the first author's name, year of publication, country in which the study was conducted, ethnicity, cancer type, control source (population-based or hospital-based), genotyping methods, matching criteria for controls, number of cases and controls, and genotype distribution of cases and controls, was extracted from each eligible study by two independent researchers (ZNJ and LX).

The quality of eligible studies in this analysis was evaluated according to the NOS [63], which contains three perspectives: selection (four scores), comparability (two scores), and exposure (three scores). The quality of each study was independently assessed by two researchers (ZNJ and LX).

### Statistical analysis

HWE was evaluated in the controls of each study using the chi-square test. The crude odds ratio (OR) and corresponding 95% confidence interval (CI) were calculated by the Z test to assess the strength of the association between genotype and cancer risk; *P* < 0.05 was considered statistically significant.

To identify the best matching genetic model for *mTOR* polymorphisms in the occurrence of malignancies, we used the methods recommended by Thakkinstian [64]. OR1, OR2, and OR3 were calculated for genotypes VV *versus* WW, WV *versus* WW, and VV *versus* WV for each polymorphism that qualified for meta-analysis to detect the existence of heterogeneity. Appropriate genetic models were then determined in terms of the relationship between the three pairwise differences:

(1) Recessive model: if OR1 = OR3≠1 and OR2 = 1.

(2) Dominant model: if OR1 = OR2≠1 and OR3 = 1.

(3) Complete over-dominant model: if OR1 = 1, OR2 = 1/OR3≠ 1.

(4) Co-dominant model: if OR1 > OR2 > 1 and OR1 > OR3 > 1, or OR1 < OR2 < 1 and OR1 < OR3 < 1.

Using the indicated genetic model collapsed the three genotypes into two groups (except in the case of a co-dominant model):

(1) If a dominant model was indicated, V carriers (VV plus WV) *versus* WW.

(2) If a recessive model was indicated, VV *versus* W carriers (WV plus WW).

(3) If a complete over-dominant model was indicated, (VV plus WW) *versus* WV.

(4) If a co-dominant model was indicated, VV *versus* WV, and VV versus WW.

Between-study heterogeneity was evaluated using the Q-statistical test and *I^2^* test [65]. The random-effects model (the DerSimonian and Laird method) [66] and fixed-effects model (the Mantel-Haenszel method) [67] were taken to calculate summary estimates of heterogeneous studies (Q test, *P* < 0.1 or *I^2^* > 50 %) and homogenous studies, respectively. Potential sources of heterogeneity were explored using sensitivity analysis carried out by removing a single study from the meta-analysis each time, or subgroup analyses with cancer types. Potential publication bias was estimated using Begg's rank correlation [68] and Egger's regression asymmetry test [69] (*P* < 0.1 was considered significant). STATA software version 11.0 (STATA, College Station, TX) was used for statistical analyses.

### SNP functional assessment

To cover as many bioinformatics web services and public databases as possible, we used two integration platforms of SNP analysis resources: the SNPinfo database [70] (http://snpinfo.niehs.nih.gov/snpinfo/snpfunc.htm) and the functional single nucleotide polymorphism (F-SNP) database [55, 71] (http://compbio.cs.queensu.ca/F-SNP/). Potential biological effects of the investigated *mTOR* SNPs were evaluated, including changes in protein coding, transcriptional regulation, splicing sites, and micro (mi)RNA-binding sites. Correlations between *mTOR* polymorphism genotypes and gene expression levels from 270 HapMap phase II and III individuals from four populations (CEU, CHB, JPT and YRI) were conducted online, using the SNPexp web tool [72] (http://tinyurl.com/snpexp).

## SUPPLEMENTARY MATERIAL TABLES


