# Supplementary material for: Genetic polymorphisms of mTOR and cancer risk: a systematic review and updated meta-analysis
Source: Oncotarget. 2016 Jul 24;7(35):57464–80. doi: 10.18632/oncotarget.10805 (PMC5302868; doi:10.18632/oncotarget.10805)
Supplement: Supplementary file 1 [file oncotarget-07-57464-s001.docx]

Appendix 1 Information about *mTOR* polymorphisms investigated in relation to cancer risk

| Polymorphisms | Location | Author | Year | Cancer type | Country | Ethnicity | Case/control | Compared genotype | OR(95%CI)/P | Adjusting/matching factors |
| --- | --- | --- | --- | --- | --- | --- | --- | --- | --- | --- |
| rs1057079 (A>G) | Exon | Slattery, M. L[[13](#_ENREF_13)] | 2010 | Colon cancer | USA | Non-Hispanic white | 1444/1841 | GA/GG vs. AA | 1.21 (1.05–1.38) | age, sex race, center |
|  |  |  |  |  |  | Hispanic or American Indian | 60/75 |  |  |  |
|  |  |  |  |  |  | African-American | 70/54 |  |  |  |
|  |  |  |  | Rectal cancer |  | Non-Hispanic white | 657/856 | GA/GG vs. AA | 1.06 (0.87–1.28) | age, sex race, center |
|  |  |  |  |  |  | Hispanic or American Indian | 63/69 |  |  |  |
|  |  |  |  |  |  | African-American | 31/44 |  |  |  |
|  |  |  |  |  |  | Asian | 40/30 |  |  |  |
|  |  |  |  |  |  |  |  |  |  |  |
|  |  | Slattery, M. L[[54](#_ENREF_54)] | 2012 | Breast cancer | USA | Non-Hispanic white | 1481/1586 | AA* | 0.71 (0.56-0.89) | age, center, BMI, parity, |
|  |  |  |  |  |  | Hispanic | 2111/2597 | AG* | 0.79 (0.61-1.02) | age at first birth, |
|  |  |  |  |  |  |  |  | GG* | 1.32 (0.80-2.17) | alcohol intake, |
|  |  | Zhu,J.H.[[20](#_ENREF_20)] | 2015 | Esophageal carcinoma | China | Asian | 1116/1117 | TC vs.TT | 1.10 (0.91–1.32) | age, sex, |
|  |  |  |  |  |  |  |  | CC vs.TT | 1.18 (0.75–1.85) | smoking, |
|  |  |  |  |  |  |  |  | CC/TC vs.TT | 1.11 (0.93–1.33) | and drinking status |
|  |  |  |  |  |  |  |  |  |  |  |
| rs2024627(C>T) | Intron | Slattery, M. L[[13](#_ENREF_13)] | 2010 | Colon cancer | USA | Non-Hispanic white | 1444/1841 | CT vs. CC | 1.17 (1.01-1.34) |  |
|  |  |  |  |  |  | Hispanic or American Indian | 60/75 | TT vs. CC | 1.02 (0.79-1.32) |  |
|  |  |  |  |  |  | African-American | 70/54 |  |  |  |
|  |  |  |  | Rectal cancer |  | Non-Hispanic white | 657/856 | CT vs. CC | 1.27 (0.88-1.85) |  |
|  |  |  |  |  |  | Hispanic or American Indian | 63/69 | TT vs. CC | 0.96 (0.78-1.17) |  |
|  |  |  |  |  |  | African-American | 31/44 |  |  |  |
|  |  |  |  |  |  | Asian | 40/30 |  |  |  |
| rs718206(A>T) | Intron | Slattery, M. L[[13](#_ENREF_13)] | 2010 | Colon cancer | USA | Non-Hispanic white | 1444/1841 | TA vs. AA | 1.17 (1.02-1.35) |  |
|  |  |  |  |  |  | Hispanic or American Indian | 60/75 | TT vs. AA | 1.03 (0.80-1.33) |  |
|  |  |  |  |  |  | African-American | 70/54 |  |  |  |
|  |  |  |  | Rectal cancer |  | Non-Hispanic white | 657/856 | TA vs. AA | 1.34 (0.93-1.95) |  |
|  |  |  |  |  |  | Hispanic or American Indian | 63/69 | TT vs. AA | 0.94 (0.77-1.16) |  |
|  |  |  |  |  |  | African-American | 31/44 |  |  |  |
|  |  |  |  |  |  | Asian | 40/30 |  |  |  |
| rs11121691(C>T) | Exon | Wang, L. E[[21](#_ENREF_21)] | 2012 | Endometrial cancer | USA | Non-Hispanic white (76.2%) | 115/230 | Additive model | P=0.346 | Not mentioned |
|  |  |  |  |  |  | African-American (8.7%) |  | TT/TC vs.CC | P=0.236 | Not mentioned |
|  |  |  |  |  |  | Mexican-American (14.8%) |  | TT vs.TC/CC | P=0.967 | Not mentioned |
|  |  |  |  |  |  |  |  |  |  |  |
| rs11121696(T>C) | Intron | Shu, X.[[22](#_ENREF_22)] | 2013 | Renal cell carcinoma | USA | Non-Hispanic white | 577/593 | Not mentioned | Not mentioned | Not mentioned |
|  |  | Lin, J.[[73](#_ENREF_73)] | 2010 | Bladder cancer | USA | Caucasian | 803/803 | not significantly associated with cancer risk | | age, sex, tobacco smoking, |
|  |  |  |  |  |  |  |  |  |  | BMI, energy intake, |
|  |  |  |  |  |  |  |  |  |  | and physical activity |
| rs1770345(A>C) | Intron | Wang, L. E***[[21](#_ENREF_21)]. | 2012 | Endometrial cancer | USA | Non-Hispanic white (76.2%) | 115/230 | / | / | / |
|  |  |  |  |  |  | African-American (8.7%) |  |  |  |  |
|  |  |  |  |  |  | Mexican-American (14.8%) |  |  |  |  |
|  |  | Shu, X.[[22](#_ENREF_22)] | 2013 | Renal cell carcinoma | USA | Non-Hispanic white | 577/593 | Not mentioned | Not mentioned | Not mentioned |
|  |  | Campa, D.[[74](#_ENREF_74)] | 2011 | Prostate cancer | Germany | Caucasian(>97%) | 815/1266 | CA vs. AA | 1.09 (0.87-1.36) | age, center |
|  |  |  |  |  |  |  |  | CC vs. AA | 1.14 (0.87-1.49) |  |
|  |  |  |  |  |  |  |  | C allele vs. A allele | 1.07 (0.93-1.22) |  |
|  |  | Lin, J.[[73](#_ENREF_73)] | 2010 | Bladder cancer | USA | Caucasian | 803/803 | not significantly associated with cancer risk | | age, sex, tobacco smoking, |
|  |  |  |  |  |  |  |  |  |  | BMI, energy intake, |
|  |  |  |  |  |  |  |  |  |  | and physical activity |
| rs12124983(C>T) | Intron | Wang, L. E.[[21](#_ENREF_21)] | 2012 | Endometrial cancer | USA | Non-Hispanic white (76.2%) | 115/230 | Additive model | P=0.105 | Not mentioned |
|  |  |  |  |  |  | African-American (8.7%) |  | TT/TC vs.CC | P=0.251 | Not mentioned |
|  |  |  |  |  |  | Mexican-American (14.8%) |  | TT vs.TC/CC | P=0.134 | Not mentioned |
|  |  | Ter-Minassian, M.[[53](#_ENREF_53)] | 2011 | Neuroendocrine tumor | USA | not given | 261/319 | Additive model | 1.51 (1.16-1.97) | age, sex and smoking |
|  |  |  |  |  |  |  |  | TT/TC vs.CC | 1.52 (1.07-2.14) |  |
|  |  |  |  |  |  |  | 235/113 | Additive model | 0.90 (0.63-1.27) | age and sex |
|  |  |  |  |  |  |  |  | TT/TC vs.CC | 0.83 (0.53-1.32) |  |
|  |  |  |  |  |  |  |  |  |  |  |
| rs1010447(G>A) | Intron | Campa, D.[[74](#_ENREF_74)] | 2011 | Prostate cancer | Germany | Caucasian(>97%) | 815/1266 | GA vs.GG | 0.87 (0.72-1.06) | age, center |
|  |  |  |  |  |  |  |  | AA vs.GG | 1.10 (0.77-1.58) |  |
|  |  |  |  |  |  |  |  | A allele vs. G allele | 0.96 (0.83-1.11) |  |
|  |  |  |  |  |  |  |  |  |  |  |
| rs1074078(G>A) | 5' | Campa, D.[[74](#_ENREF_74)] | 2011 | Prostate cancer | Germany | Caucasian(>97%) | 815/1266 | GA vs.GG | 0.85 (0.70-1.03) | age, center |
|  | upstream | |  |  |  |  |  | AA vs.GG |  |  |
|  |  |  |  |  |  |  |  | A allele vs. G allele | 0.90 (0.79-1.03) |  |
|  |  |  |  |  |  |  |  |  |  |  |
| rs12732063(G>A) | Intron | Campa, D.[[74](#_ENREF_74)] | 2011 | Prostate cancer | Germany | Caucasian(>97%) | 815/1266 | GA vs.GG | 1.18 (0.85-1.63) | age, center |
|  |  |  |  |  |  |  |  | AA vs.GG | 2.68 (0.44-16.32) |  |
|  |  |  |  |  |  |  |  | A allele vs. G allele | 1.22 (0.90-1.66) |  |
|  |  |  |  |  |  |  |  |  |  |  |
| rs1034528(G>C) | Intron | Li, Q.[[30](#_ENREF_30)] | 2013 | Prostate cancer | China | Asian | 1004/1051 | GC vs.GG | 1.31 (1.08–1.59) | age, smoking, BMI |
|  |  |  |  |  |  |  |  | CC vs.GG | 1.09 (0.66–1.79) |  |
|  |  |  |  |  |  |  |  | CC/GC vs.GG | 1.29 (1.07–1.55) |  |
|  |  |  |  |  |  |  |  | CC vs. GC/GG | 1.00 (0.61–1.64) |  |
|  |  |  |  |  |  |  |  | Additive model | 1.21 (1.03–1.42) |  |
|  |  | Wang,M.#[[16](#_ENREF_16)] | 2014 | Gastric cancer | China | Asian | 1123/1113 | CC/GC vs. GG | 1.21(1.01-1.46) | age,sex |
|  |  | Wang,M.Y.[[18](#_ENREF_18)] | 2015 | Gastric cancer | China | Asian | 1002/1003 | GC vs.GG | 1.27 (1.05–1.55) | age, sex, |
|  |  |  |  |  |  |  |  | CC vs.GG | 0.80 (0.46–1.39) | smoking, |
|  |  |  |  |  |  |  |  | CC/GC vs.GG | 1.23 (1.02–1.48) | and drinking status |
|  |  |  |  |  |  |  |  | Additive model | 0.121 |  |
| rs17036508(T>C) | Intron | Li, Q.[[30](#_ENREF_30)] | 2013 | Prostate cancer | China | Asian | 1004/1051 | TC vs.TT | 1.23 (0.99–1.52) | age, smoking, BMI |
|  |  |  |  |  |  |  |  | CC vs.TT | 0.94 (0.49–1.77) |  |
|  |  |  |  |  |  |  |  | CC/TC vs.TT | 1.20 (0.98–1.48) |  |
|  |  |  |  |  |  |  |  | CC vs.TC/TT | 0.89 (0.47–1.69) |  |
|  |  |  |  |  |  |  |  | Additive model | 1.15 (0.96–1.38) |  |
|  |  | Wang,M.Y.[[18](#_ENREF_18)] | 2015 | Gastric cancer | China | Asian | 1002/1003 | TC vs.TT | 1.22 (0.99–1.52) | age, sex, |
|  |  |  |  |  |  |  |  | CC vs.TT | 1.06 (0.57–2.00) | smoking, |
|  |  |  |  |  |  |  |  | CC/TC vs.TT | 1.21 (0.99–1.49) | and drinking status |
|  |  |  |  |  |  |  |  | Additive model | 0.098 |  |
|  |  |  |  |  |  |  |  |  |  |  |
|  |  |  |  |  |  |  |  |  |  |  |
| rs3806317(A>G) | Intron | Li, Q.[[30](#_ENREF_30)] | 2013 | Prostate cancer | China | Asian | 1004/1051 | AG vs. AA | 0.93 (0.76–1.15) | age, smoking, BMI |
|  |  |  |  |  |  |  |  | GG vs. AA | 0.61 (0.29–1.25) |  |
|  |  |  |  |  |  |  |  | GG/AG vs. AA | 0.91 (0.74–1.11) |  |
|  |  |  |  |  |  |  |  | GG vs.AG/AA | 0.62 (0.30–1.27) |  |
|  |  |  |  |  |  |  |  | Additive model | 0.89 (0.74–1.07) |  |
|  |  | Wang,M.Y.[[18](#_ENREF_18)] | 2015 | Gastric cancer | China | Asian | 1002/1003 | AG vs. AA | 1.25 (1.02–1.53) | age, sex, |
|  |  |  |  |  |  |  |  | GG vs. AA | 0.99 (0.54–1.84) | smoking, |
|  |  |  |  |  |  |  |  | GG/AG vs. AA | 1.22 (1.00–1.49) | and drinking status |
|  |  |  |  |  |  |  |  | Additive model | 0.087 |  |
| rs11585553(G>A) | Intron | Lin, J.[[73](#_ENREF_73)] | 2010 | Bladder cancer | USA | Caucasian | 803/803 | not significantly associated with cancer risk | | age, sex, tobacco smoking, |
|  |  |  |  |  |  |  |  |  |  | BMI, energy intake, |
|  |  |  |  |  |  |  |  |  |  | and physical activity |
|  |  |  |  |  |  |  |  |  |  |  |
| rs1064261(A>G) | Exon | Zhu,J.H.[[20](#_ENREF_20)] | 2015 | Esophageal carcinoma | China | Asian | 1116/1117 | GA vs. AA | 1.22 (0.96–1.55) | age, sex, |
|  |  |  |  |  |  |  |  | GG vs. AA | 0.87 (0.24–3.22) | smoking, |
|  |  |  |  |  |  |  |  | GA/GG vs. AA | 1.21 (0.96–1.53) | and drinking status |
|  |  | Ter-Minassian, M.[[53](#_ENREF_53)] | 2011 | Neuroendocrine tumor | USA | not given | 261/319 | Additive model | 1.45 (1.11-1.90) | age, sex and smoking |
|  |  |  |  |  |  |  |  | GA/GG vs. AA | 1.42 (1.01-2.01) |  |
|  |  |  |  |  |  |  | 235/113 | Additive model | 0.99 (0.71-1.37) | age and sex |
|  |  |  |  |  |  |  |  | GA/GG vs. AA | 0.84 (0.53-1.34) |  |
|  |  | Piao, Y.[[17](#_ENREF_17)] | 2015 | Gastric cancer | China | Asian | 483/673 | TC vs. TT | 0.96(0.67-1.37) | sex, age |
|  |  |  |  |  |  |  |  | CC vs. TT | 1.26(0.35-4.51) | H.pylori infection status |
|  |  |  |  |  |  |  |  |  |  |  |
| rs12116957(G>T) | Intron | Cheng,T.Y.D.#[[27](#_ENREF_27)] | 2014 | Breast cancer | USA | European American | 658/649 |  | P=0.09 | age, family history, BMI |
|  |  |  |  |  |  |  |  |  |  | education, history of benign |
|  |  |  |  |  |  | African-American | 621/744 |  | P>0.1 | breast disease, cigarette smoking, |
|  |  |  |  |  |  |  |  |  |  | proportion of European ancestry, |
|  |  |  |  |  |  |  |  |  |  |  |
| rs12125777(C>T) | Intron | Cheng,T.Y.D.#[[27](#_ENREF_27)] | 2014 | Breast cancer | USA | European American | 658/649 | TT/TC vs CC | 1.71(1.10-2.66) | age, family history, BMI |
|  |  |  |  |  |  |  |  |  |  | education, history of benign |
|  |  |  |  |  |  | African-American | 621/744 | TT/TC vs CC | P>0.1 | breast disease, cigarette smoking, |
|  |  |  |  |  |  |  |  |  |  | proportion of European ancestry, |
|  |  |  |  |  |  |  |  |  |  |  |
| rs2536(T>C) | 3'-UTR | Zhu, M. L[[19](#_ENREF_19)] | 2013 | Esophageal carcinoma | China | Asian | 1123/1121 | TC vs.TT | 1.12 (0.88–1.43) | age, sex, BMI, |
|  |  |  |  |  |  |  |  | CC vs.TT | 1.21 (0.41–3.57) | smoking , |
|  |  |  |  |  |  |  |  | CC/TC vs.TT | 1.12 (0.88–1.43) | and drinking status |
|  |  |  |  |  |  |  |  | CC vs.TC/TT | 1.19 (0.40–3.51) |  |
|  |  |  |  |  |  |  |  |  |  |  |
|  |  | Li, Q.[[30](#_ENREF_30)] | 2013 | Prostate cancer | China | Asian | 1004/1051 | TC vs.TT | 1.45 (1.15–1.84) | age, smoking, BMI |
|  |  |  |  |  |  |  |  | CC vs.TT | 0.88 (0.35–2.25) |  |
|  |  |  |  |  |  |  |  | CC/TC vs.TT | 1.42 (1.13–1.78) |  |
|  |  |  |  |  |  |  |  | CC vs.TC/TT | 0.83 (0.33–2.12) |  |
|  |  |  |  |  |  |  |  | Additive model | 1.34 (1.08–1.66) |  |
|  |  | He, J.[[14](#_ENREF_14)] | 2013 | Gastric cancer | China | Asian | 1125/1196 | TC vs.TT | 1.15 (0.91–1.44) | age, sex, |
|  |  |  |  |  |  |  |  | CC vs.TT | 1.26 (0.45–3.52) | smoking, |
|  |  |  |  |  |  |  |  | CC/TC vs.TT | 1.15 (0.92–1.44) | and drinking status |
|  |  | Huang, L.[[23](#_ENREF_23)] | 2012 | ALL | China | Asian | 417/554 | TC vs.TT | 0.67(0.46-0.96) | age, sex, |
|  |  |  |  |  |  |  |  | CC vs.TT | 2.43(0.56-10.63) | parental smoking |
|  |  |  |  |  |  |  |  | CC/TC vs.TT | 0.71(0.50-1.02) | and drinking status |
|  |  | Chen, J.[[29](#_ENREF_29)] | 2012 | Prostate cancer | China | Asian | 666/708 | TC vs.TT | 0.82(0.61–1.11) | age, smoking, |
|  |  |  |  |  |  |  |  | CC vs.TT | 1.26(0.33–4.84) | drinking status |
|  |  |  |  |  |  |  |  |  |  | and family history of cancer |
|  |  | Cao, Q.[[10](#_ENREF_10)] | 2012 | Renal cell cancer | China | Asian | 710/760 | TC vs.TT | 0.77 (0.58–1.03) | age, sex, |
|  |  |  |  |  |  |  |  | CC vs.TT | 0.84 (0.19–3.61) | smoking, drinking status |
|  |  |  |  |  |  |  |  | CC/TC vs.TT | 0.77 (0.58–1.02) | diabetes and hypertension |
|  |  | Mao, L. Q.[[49](#_ENREF_49)] | 2013 | Hepatocellular | China | Asian | 1048/1052 | TC vs.TT | 1.03(0.74-1.44) | age, sex, smoking, |
|  |  |  |  | carcinoma |  |  |  | CC vs.TT | 0.46(0.16-1.36) | drinking status |
|  |  |  |  |  |  |  |  | CC/TC vs.TT | 0.98(0.71-1.35) | ethnicity, and HBV infection |
|  |  | Zhang,H.Y.^[[28](#_ENREF_28)] | 2014 | Meningioma | China | Asian | 199/264 | not significantly associated with meningioma risk | | |
|  |  | Cheng,T.Y.D.#[[27](#_ENREF_27)] | 2014 | Breast cancer | USA | European American | 658/649 | CC/TC vs.TT | P=0.08 | age, family history, BMI |
|  |  |  |  |  |  |  |  |  |  | education, history of benign |
|  |  |  |  |  |  | African-American | 621/744 | CC/TC vs.TT | P>0.1 | breast disease, cigarette smoking, |
|  |  |  |  |  |  |  |  |  |  | proportion of European ancestry, |
| rs2295080(T>G) | Promoter | Slattery, M. L[[13](#_ENREF_13)] | 2010 | Colon cancer | USA | Non-Hispanic white | 1444/1841 | GT vs. TT | 1.19 (1.03-1.38) |  |
|  | region |  |  |  |  | Hispanic or American Indian | 60/75 | GG vs. TT | 1.21 (0.95-1.53) |  |
|  |  |  |  |  |  | African-American | 70/54 |  |  |  |
|  |  |  |  | Rectal cancer |  | African-American | 657/856 | GT vs. TT | 1.27 (0.91-1.78) |  |
|  |  |  |  |  |  | African-American | 63/69 | GG vs. TT | 1.06 (0.86-1.30) |  |
|  |  |  |  |  |  | African-American | 31/44 |  |  |  |
|  |  |  |  |  |  | African-American | 40/30 |  |  |  |
|  |  | Slattery, M. L[[54](#_ENREF_54)] | 2012 | Breast cancer | USA | African-American | 1481/1586 | TT** | 0.54 (0.39-0.74) | age, center, BMI, parity, |
|  |  |  |  |  |  | African-American | 2111/2597 | TG** | 0.65 (0.46-0.91) | age at first birth, |
|  |  |  |  |  |  |  |  | GG** | 1.36 (0.71-2.58) | alcohol intake, |
|  |  | Wang, L. E[[21](#_ENREF_21)] | 2012 | Endometrial cancer | USA | Non-Hispanic white (76.2%) | 115/230 | Additive model | P=0.319 | Not mentioned |
|  |  |  |  |  |  | African-American (8.7%) |  | GG/TG vs.TT | P=0.824 | Not mentioned |
|  |  |  |  |  |  | Mexican-American (14.8%) |  | GG vs.TG/TT | P=0.105 | Not mentioned |
|  |  | Xu, M.[[15](#_ENREF_15)] | 2013 | Gastric cancer | China | Asian | 753/854 | TG vs.TT | 0.83 (0.67–1.02) | age, sex |
|  |  |  |  |  |  |  |  | GG vs.TT | 0.49 (0.30–0.80) |  |
|  |  |  |  |  |  |  |  | GG/TG vs.TT | 0.78 (0.64–0.96) |  |
|  |  |  |  |  |  |  |  | G allele vs. T allele | 0.77 (0.65–0.92) |  |
|  |  | Li, Q.[[30](#_ENREF_30)] | 2013 | Prostate cancer | China | Asian | 1004/1051 | TG vs.TT | 0.77 (0.64–0.93) | age, smoking, BMI |
|  |  |  |  |  |  |  |  | GG vs.TT | 0.73 (0.48–1.12) |  |
|  |  |  |  |  |  |  |  | GG/TG vs.TT | 0.76 (0.64–0.92) |  |
|  |  |  |  |  |  |  |  | GG vs.TG/TT | 0.8 (0.52–1.22) |  |
|  |  |  |  |  |  |  |  | Additive model | 0.80 (0.69–0.94) |  |
|  |  | Huang, L.[[23](#_ENREF_23)] | 2012 | ALL | China | Asian | 417/554 | TG vs.TT | 1.01(0.75-1.35) | age, sex, |
|  |  |  |  |  |  |  |  | GG vs.TT | 1.49(0.78-2.85) | parental smoking |
|  |  |  |  |  |  |  |  | GG/TG vs.TT | 1.05(0.80-1.39) | and drinking status |
|  |  | Chen, J.[[29](#_ENREF_29)] | 2012 | Prostate cancer | China | Asian | 666/708 | TG vs.TT | 0.77(0.61–0.98) | age, smoking, |
|  |  |  |  |  |  |  |  | GG vs.TT | 0.74(0.44–1.24) | drinking status |
|  |  |  |  |  |  |  |  | GG/TG vs.TT | 0.77(0.62–0.96) | and family history of cancer |
|  |  |  |  |  |  |  |  | G allele vs. T allele | 0.85(0.74–0.98) | Allele-specific ORs were not adjusted |
|  |  | Cao, Q.[[10](#_ENREF_10)] | 2012 | Renal cell cancer | China | Asian | 710/760 | TG vs.TT | 0.81(0.68–0.97) | age, sex, |
|  |  |  |  |  |  |  |  | GG vs.TT | 0.86(0.55–1.37) | smoking, drinking status |
|  |  |  |  |  |  |  |  | GG/TG vs.TT | 0.74 (0.59–0.91) | diabetes and hypertension |
|  |  | Zhu,J.H.[[20](#_ENREF_20)] | 2015 | Esophageal carcinoma | China | Asian | 1116/1117 | TG vs.TT | 1.13 (0.94–1.36) | age, sex, |
|  |  |  |  |  |  |  |  | GG vs.TT | 1.12 (0.73–1.71) | smoking, |
|  |  |  |  |  |  |  |  | GG/TG vs.TT | 1.13 (0.95–1.36) | and drinking status |
|  |  | Xu,M.[[25](#_ENREF_25)] | 2015 | Colon cancer | China | Asian | 374/777 | GG/TG vs.TT | 0.60 (0.46–0.79) | age,sex |
|  |  |  |  | Rectum cancer |  |  | 335/777 | GG/TG vs.TT | 0.91 (0.70–1.19) |  |
|  |  |  |  | Intestine cancer |  |  | 28/777 | GG/TG vs.TT | 1.56 (0.72–3.34) |  |
|  |  | Wang,M.Y.[[18](#_ENREF_18)] | 2015 | Gastric cancer | China | Asian | 1002/1003 | TG vs.TT | 1.18 (0.98–1.42) | age, sex, |
|  |  |  |  |  |  |  |  | GG vs.TT | 1.04 (0.66–1.63) | smoking, |
|  |  |  |  |  |  |  |  | GG/TG vs.TT | 1.17 (0.97–1.39) | and drinking status |
|  |  |  |  |  |  |  |  | Additive model | 0.163 |  |
|  |  | Zhao, P.[[24](#_ENREF_24)] | 2015 | ALL | China | Asian | 180/296 | GG vs.TT | 3.180(1.416–7.143) |  |
|  |  |  |  |  |  |  |  | G vs. T | 1.456(1.052–2.015) |  |
|  |  |  |  | AML | China | Asian | 180/296 | GG vs.TT | 3.204(1.109–9.253) |  |
|  |  |  |  |  |  |  |  | G vs. T | 1.294(0.792–2.115) |  |
| rs1883965(G>A) | Intron | Zhu, M. L[[19](#_ENREF_19)] | 2013 | Esophageal carcinoma | China | Asian | 1123/1121 | GA vs.GG | 1.27 (1.01–1.60) | age, sex, BMI, |
|  |  |  |  |  |  |  |  | AA vs.GG | 1.02 (0.33–3.16) | smoking , |
|  |  |  |  |  |  |  |  | AA/GA vs.GG | 1.26 (1.01–1.58) | and drinking status |
|  |  |  |  |  |  |  |  | AA vs.GA/GG | 0.98 (0.32–3.03) |  |
|  |  | Li, Q.[[30](#_ENREF_30)] | 2013 | Prostate cancer | China | Asian | 1004/1051 | GA vs.GG | 1.06 (0.83–1.35) | age, smoking, BMI |
|  |  |  |  |  |  |  |  | AA vs.GG | 1.33 (0.48–3.70) |  |
|  |  |  |  |  |  |  |  | AA/GA vs.GG | 1.07 (0.84–1.36) |  |
|  |  |  |  |  |  |  |  | AA vs.GA/GG | 1.32 (0.47–3.66) |  |
|  |  |  |  |  |  |  |  | Additive model | 1.08 (0.86–1.34) |  |
|  |  | He, J.[[14](#_ENREF_14)] | 2013 | Gastric cancer | China | Asian | 1125/1196 | GA vs.GG | 1.26 (1.00–1.59) | age, sex, |
|  |  |  |  |  |  |  |  | AA vs.GG | 1.85 (0.67–5.16) | smoking, |
|  |  |  |  |  |  |  |  | AA/GA vs.GG | 1.28 (1.03–1.61) | and drinking status |
|  |  | Mao, L. Q.[[49](#_ENREF_49)] | 2013 | Hepatocellular carcinoma | China | Asian | 1048/1052 | GA vs.GG | 1.10(0.77-1.56) | age, sex, smoking, |
|  |  |  |  |  |  |  |  | AA vs.GG | 0.35(0.04-2.84) | drinking status |
|  |  |  |  |  |  |  |  | AA/GA vs.GG | 1.06(0.75-1.50) | ethnicity, and HBV infection |
|  |  | Zhang,H.Y.^[[28](#_ENREF_28)] | 2014 | Meningioma | China | Asian | 199/264 | not significantly associated with cancer risk | | |

Abbreviations: ALL, acute lymphoblastic leukemia; AML, acute myeloid leukemia.

* Indicates genotypes in women with high Native American ancestry, compared with wild-type AA genotype in women with low Native American ancestry.

** Indicates genotypes in postmenopausal women with high Native American ancestry, compared with wild-type TT genotype in postmenopausal women with low Native American ancestry.

*** Not in HWE

# Abstract

^ Chi-square test

PubMed search strategy

(((("Polymorphism, Single Nucleotide"[Mesh]) OR "Polymorphism, Genetic"[Mesh]) OR (((Polymorphism*[Title/Abstract]) OR Variant*[Title/Abstract]) OR Mutation*[Title/Abstract]))) AND (((((((("Probability"[Mesh]) OR "Disease Susceptibility"[Mesh]) OR "Risk"[Mesh]) OR (((Risk*[Title/Abstract]) OR Susceptibilit*[Title/Abstract]) OR Probabilit*[Title/Abstract]))) AND ((((((((cancer*[Title/Abstract]) OR carcinoma*[Title/Abstract]) OR neoplas*[Title/Abstract]) OR tumor*[Title/Abstract]) OR tumour*[Title/Abstract]) OR malignan*[Title/Abstract])) OR "Neoplasms"[Mesh]))) AND ((((((Mtor[Title/Abstract]) OR Frap*[Title/Abstract]) OR RAFT1[Title/Abstract]) OR RAPT1[Title/Abstract])) OR "MTOR protein, human" [Supplementary Concept]))
